# Supplementary material for: The SWI/SNF chromatin remodeling factor DPF3 regulates metastasis of ccRCC by modulating TGF-β signaling
Source: Nat Commun. 2022 Aug 9;13:4680. doi: 10.1038/s41467-022-32472-0 (PMC9363427; doi:10.1038/s41467-022-32472-0)
Supplement: Supplementary file 1 — Supplementary Information [file 41467_2022_32472_MOESM1_ESM.pdf]

**Supplementary information to:**

# The SWI/SNF chromatin remodeling factor DPF3 regulates metastasis of ccRCC by modulating TGF-β signaling

Huanhuan Cui[1,2,3,*], Hongyang Yi[2,#], Hongyu Bao[2,4,#], Ying Tan[2,#], Chi Tian[2,#], Xinyao Shi[2], Diwen Gan[2], Bin Zhang[5], Weizheng Liang[2], Rui Chen[2], Qionghua Zhu[1,2], Liang Fang[1,2,3], Xin Gao[5], Hongda Huang[2,4], Ruijun Tian[6], Silke R. Sperling[7], Yuhui Hu[1,2], Wei Chen[1,2,3,*]

1. Shenzhen Key Laboratory of Gene Regulation and Systems Biology, School of Life Sciences, Southern University of Science and Technology, Shenzhen 518005, China
2. Department of Biology, School of Life Sciences, Southern University of Science and Technology, Shenzhen 518005, China
3. Academy for Advanced Interdisciplinary Studies, Southern University of Science and Technology, Shenzhen 518005, China
4. Key Laboratory of Molecular Design for Plant Cell Factory of Guangdong Higher Education Institutes, School of Life Sciences, Southern University of Science and Technology, Shenzhen 518005, China
5. Computational Bioscience Research Center, Computer, Electrical and Mathematical Sciences and Engineering Division, King Abdullah University of Science and Technology (KAUST), Thuwal 23955-6900, Saudi Arabia
6. Department of Chemistry, School of Science, Southern University of Science and Technology, Shenzhen 518005, China
7. Cardiovascular Genetics, Charité-Universitätsmedizin Berlin, 13125 Berlin, Germany

* Corresponding authors: cuihh@sustech.edu.cn, chenw@sustech.edu.cn

[#] These authors contributed equally

**Supplementary Fig. 1**

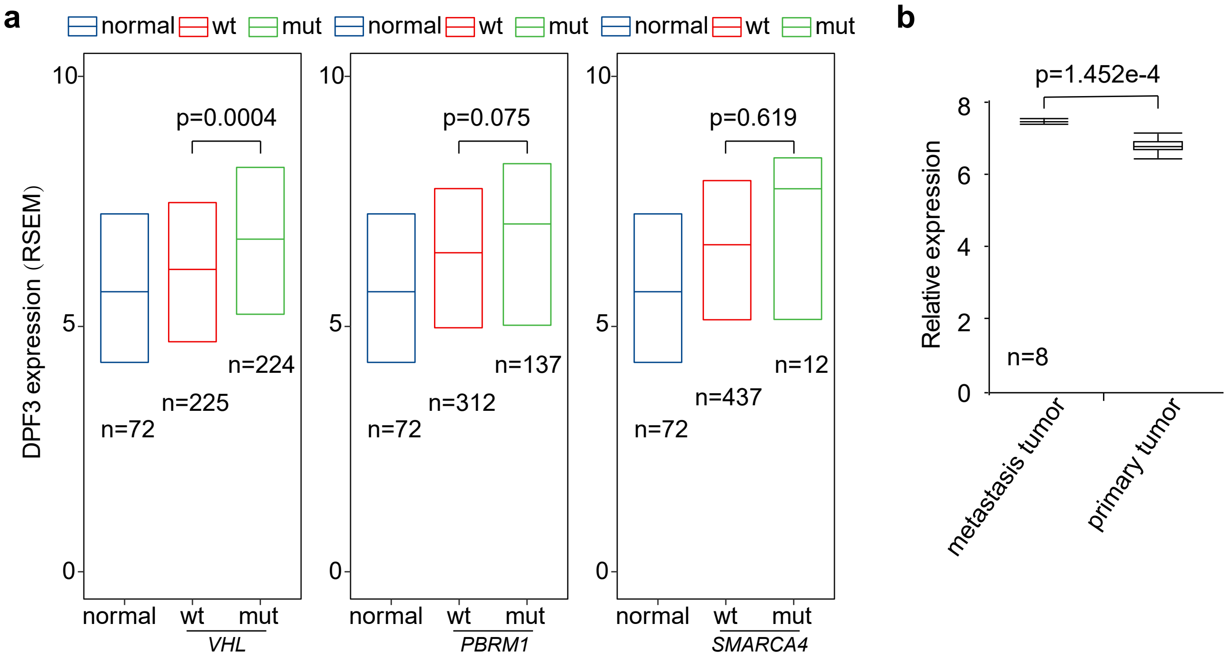

**Supplementary Fig. 1│Upregulation of DPF3 in ccRCC, related to Fig. 1. a**, Expression analysis of *DPF3* in normal kidney tissue (n=72 individuals) and ccRCC patients with and without *VHL*, *PBRM1* or *SMARCA4* mutations. Results represent median expression levels with 25% and 75% quartile. Y axis represents the expression values of expectation-maximization (RSEM). Statistical significance was estimated using a two-sided Wilcoxon test. **b**, *DPF3* expression in primary and matched metastatic tumors (n=8 individuals). Results represent median expression levels with 25% and 75% quartile. Statistical significance was estimated using a two-sided Wilcoxon test. The y-axis represents relative *DPF3* expression level, while x-axis represents tumor type. The *DPF3* expression was plotted for primary and metastasis tumor separately (http://hcmdb.i-sanger.com/index).

## Supplementary Fig. 2

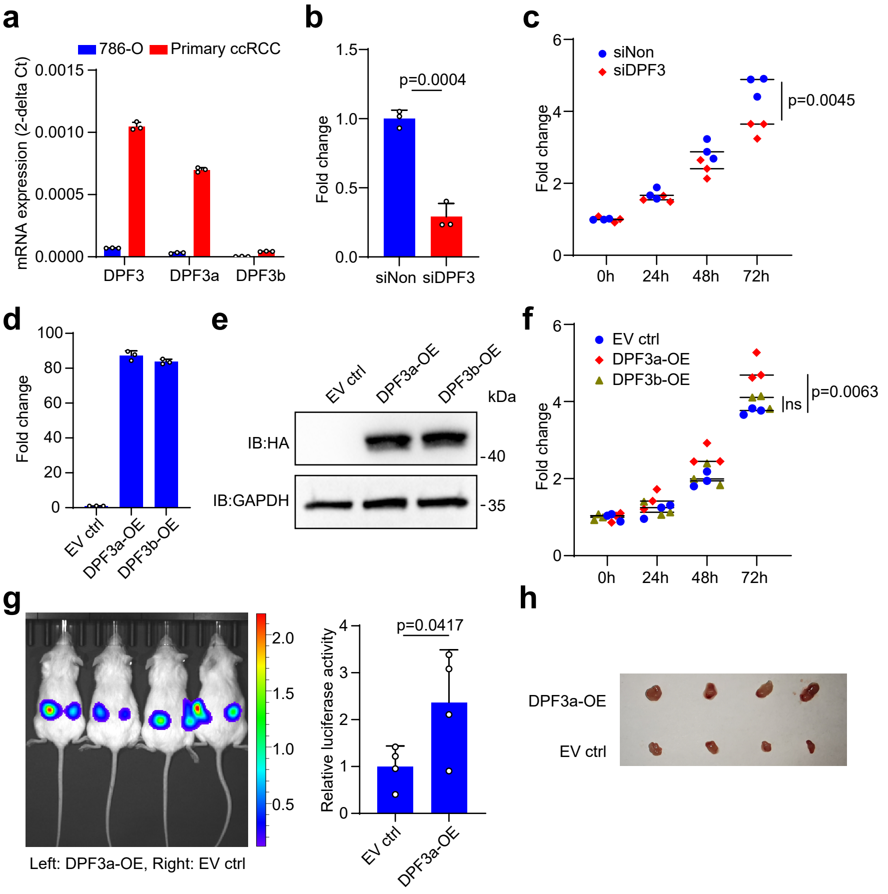

**Supplementary Fig. 2│Perturbation of DPF3 affects cell proliferation and migration, related to Fig. 2. a**, Expression of *DPF3* mRNA in 786-O cells and primary ccRCC cells. qPCR was performed on samples obtained from different cells. Expression values were normalized to the housekeeping gene *GAPDH*. Data are presented as mean values +/- SD (n=3 independent experiments). **b**, Knockdown efficiency of *DPF3* in primary ccRCC cells was analyzed by qPCR. Data are presented as mean values +/- SD (n=3 independent experiments). Statistical significance was estimated using a two-sided student t-test. **c**, Cell proliferation analysis of 786-O cells after *DPF3* knockdown using CCK8 assay. The results are presented as the fold change of mean optical density (OD) at 450 nm relative to 0h. Statistical significance was estimated using a two-sided student t-test (n=3 independent experiments). **d&e**, *DPF3* expression at mRNA level and protein level in 786-O cells after DPF3a or DPF3b overexpression, respectively. The results represent three independent biological replicates. **f**, Cell proliferation analysis of 786-O cells after DPF3a or DPF3b overexpression using CCK8 assay. Statistical significance was estimated using a two-sided student t-test (n=3 independent experiments). **g**, 786-O cells with and without DPF3a overexpression were subjected to mouse xenograft assays by orthotopic injection. Tumor cells expressing luciferase were visualized with D-Luciferin as substrate using an *in vivo* imaging system. Total luminescence intensity was quantified. Data are presented as mean values +/- SD and statistical significance was estimated using a paired two-sided student t-test (n=4 animals). **h**, Tumor tissues were dissected from mice. Source data are provided in the Source Data file.

## Supplementary Fig. 3

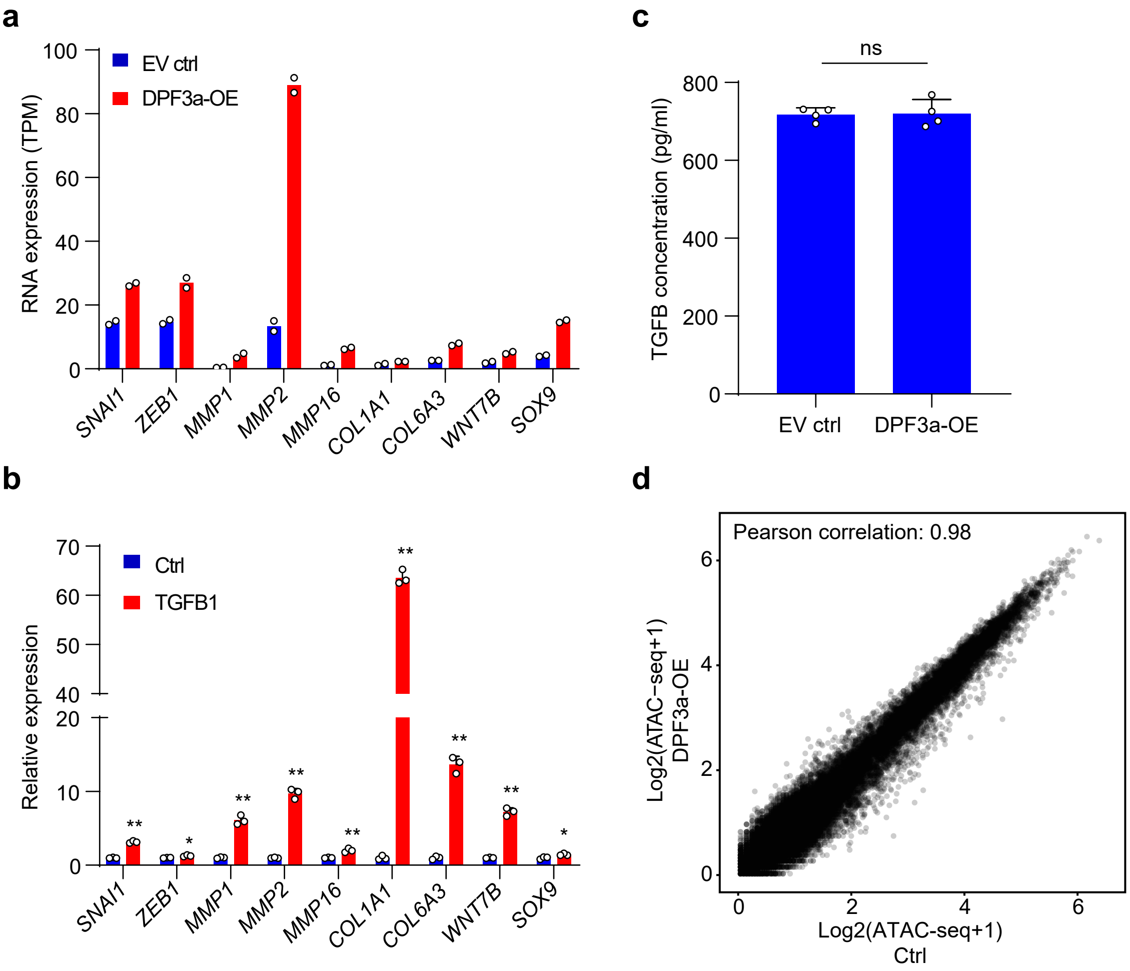

**Supplementary Fig. 3│Impacts of DPF3 overexpression on transcriptome and chromatin accessibility, related to Fig. 3. a**, Expression levels of cell migration related genes in 786-O cell with and without DPF3a overexpression. Y axis represents the expression values of transcripts per million (TPM). Data are presented as mean values (n=2 independent RNA-seq). **b**, Expression levels of DPF3a target genes were analyzed in 786-O cells after TGFB1 treatment. Statistical significance was estimated using a two-sided student t-test (n=3 independent experiments, *$p$<0.05, **$p$<0.01). $p_{SNAI1}$=0.000023, $p_{ZEB1}$=0.014, $p_{MMP1}$=0.000143, $p_{MMP2}$=0.000026, $p_{MMP16}$=0.00273, $p_{COL1A1}$<0.00001, $p_{COL6A3}$=0.000041, $p_{WNT7B}$=0.000036, $p_{SOX9}$=0.028. **c**, Levels of TGFB1 in the culture medium were measured using ELISA assay (n=4 independent experiments). **d**, Scatter plot comparing ATAC-seq readouts at promoter of all genes in 786-O cells with and without DPF3a overexpression. Pearson's correlation test was performed. Source data are provided in the Source Data file.

**Supplementary Fig. 4**

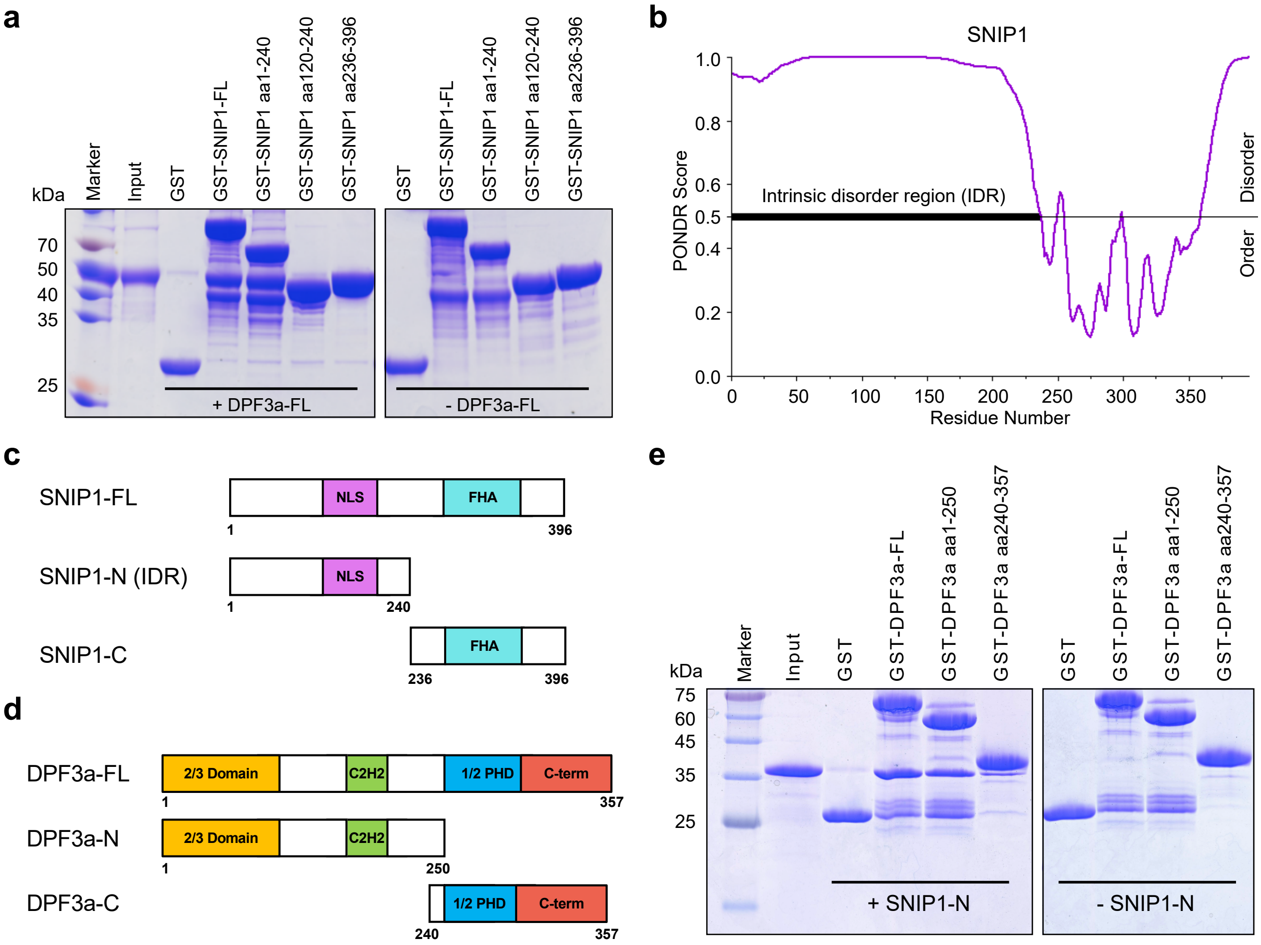

**Supplementary Fig. 4│DPF3a interacts with SNIP1, related to Fig. 4. a**, SDS-polyacrylamide gel electrophoresis of GST pulldown experiments performed with different GST-SNIP1 mutants and recombinant DPF3a. Three independent experiments were performed and similar results were obtained. **b**, Prediction of disorder tendency of human SNIP1 sequence with PONDR-VSL2 (http://www.pondr.com/). **c&d**, Schematic diagram of the SNIP1 and DPF3a constructs with GST, Flag or HA tag used in the experiments. Numbers indicate the position of amino acid. **e**, SDS-polyacrylamide gel electrophoresis of GST pulldown experiments performed with different GST-DPF3a mutants and recombinant SNIP1 N-terminus. Three independent experiments were performed and similar results were obtained. Source data are provided in the Source Data file.

**Supplementary Fig. 5**

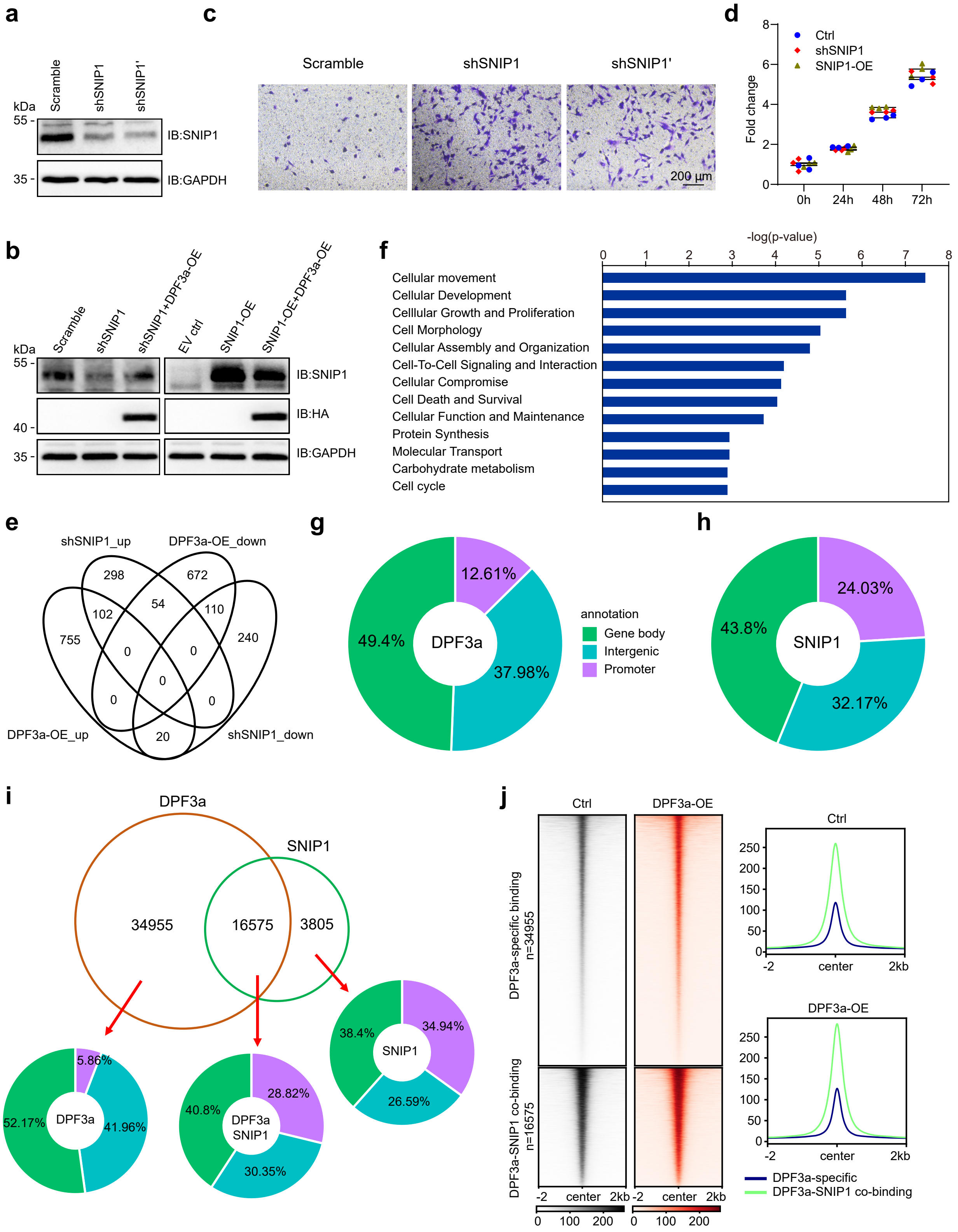

**Supplementary Fig. 5 | Regulation of cell migration related genes by SNIP1 and DPF3, related to Fig. 5. a&b**, SNIP1 and DPF3a expression at protein level in 786-O cells after DPF3a and/or SNIP1 perturbation was analyzed by immunoblotting (IB). Three independent experiments were performed and similar results were obtained. **c**, The migration ability of 786-O cells with SNIP1 knockdown was measured using Transwell assay. Three independent experiments were performed and similar results were obtained. **d**, Cell proliferation analysis of 786-O cells after SNIP1 knockdown and overexpression using CCK8 assay. The results are presented as the fold change of mean optical density (OD) at 450 nm relative to 0h (n=3 independent experiments). **e**, Overlap of differentially expressed genes after DPF3a overexpression and SNIP1 knockdown. The results represent two independent biological replicates of RNA-seq. **f**, The "Diseases & Functions" analysis of 212 genes co-regulated by SNIP1 knockdown and DPF3a overexpression using Ingenuity Pathway Analysis (IPA) software (QIAGEN). The results represent two independent biological replicates of RNA-seq. **g&h**, Distribution of DPF3a and SNIP1 binding at promoter, genebody and intergenic region in 786-O cells. The results represent two independent biological replicates of ChIP-seq. **i**, Venn diagram showing the number of overlapped ChIP-peaks between DPF3a (51,530) and SNIP1 (22,791) in 786-O cells. **j**, Aggregate plots showing the normalized read density of ATAC-seq for the 34,955 DPF3a specific ChIP-peaks and the 16,575 DPF3a and SNIP1 co-binding ChIP-peaks in 786-O cells with and without DPF3a overexpression. Source data are provided in the Source Data file.

## Supplementary Fig. 6

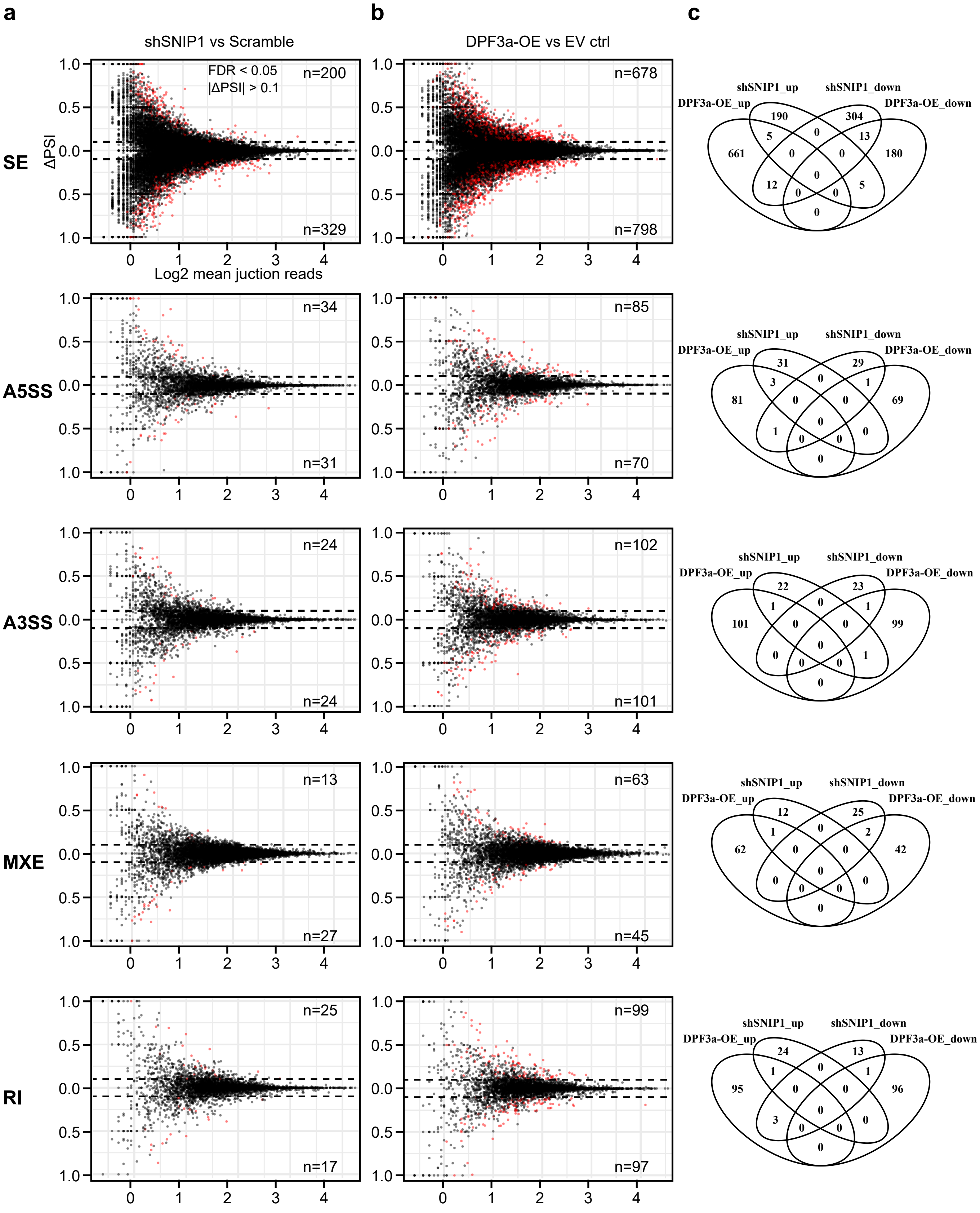

**Supplementary Fig. 6│Impact of DPF3a overexpression and SNIP1 knockdown on splicing, related to Fig. 5. a**, MA plots comparing the PSI (Percent Spliced In) values between SNIP1 knockdown and control samples in 5 different types of splicing events. The x-axis and y-axis represent Log2 (mean junction reads) and ΔPSI, respectively. SE: skipped exon; A5SS: alternative 5' splice site; A3SS: alternative 3' splice site; MXE: mutually exclusive exons; RI: retained intron. **b**, MA plots comparing the PSI values between DPF3a overexpression and control samples. **c**, Overlap of differentially spliced events after DPF3a overexpression and SNIP1 knockdown. **a-c** The results represent two independent biological replicates of RNA-seq.

**Supplementary Fig. 7**

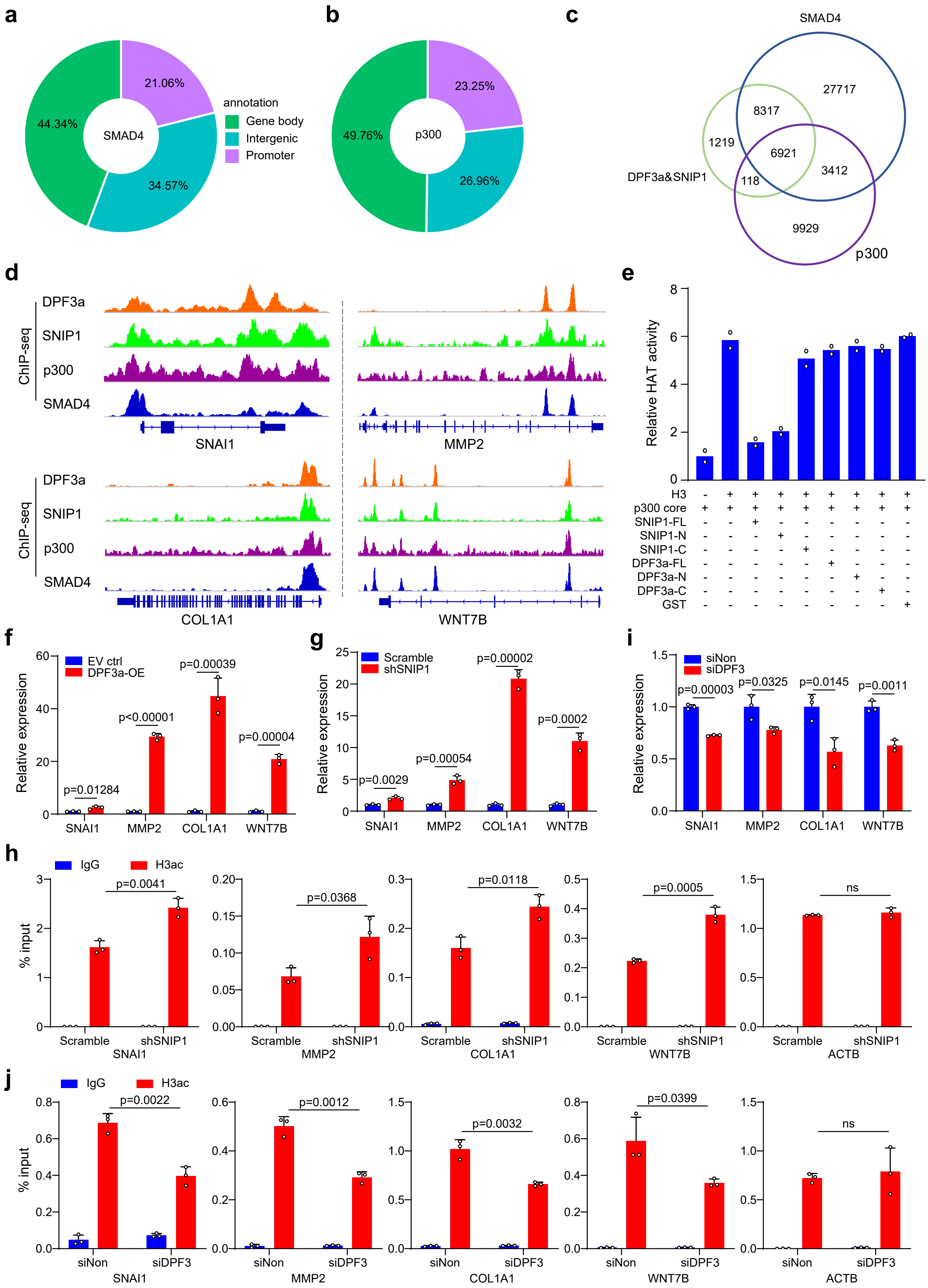

**Supplementary Fig. 7│DPF3a and SNIP1 interaction attenuates the inhibitory effect of SNIP1 on p300 HAT Activity, related to Fig. 6. a&b**, Distribution of SMAD4 and p300 binding at promoter, genebody and intergenic region in 786-O cells. **c**, Venn diagram showing the number of overlapped ChIP-peaks of DPF3a&SNIP (16,575), SMAD4 (46,367) and p300 (20,380) in 786-O cells. **d**, Genome browser view of ChIP-seq normalized read counts at *SNAI1*, *MMP2*, *COL1A1* and *WNT7B* loci. DPF3a, SNIP1 SMAD4 and p300 ChIP-peaks were perfectly overlapped at the promoter or intronic regions of the four selected genes. **e**, *In vitro* HAT activity assays of p300 in the presence of recombinant full-length, N-terminus and C-terminus of SNIP1 as well as full-length, N-terminus and C-terminus of DPF3a. Data are presented as mean values (n=2 independent experiments). **f&g**, The relative mRNA expression of *SNAI1*, *MMP2*, *COL1A1* and *WNT7B* were measure by qPCR after DPF3 overexpression and SNIP1 knockdown in 786-O cells. Data are presented as mean values +/- SD (n=3 independent experiments). Statistical significance was estimated using a two-sided student t-test. **h**, Knockdown of SNIP1 enhances local histone acetylation at specific loci in 786-O cells. Pan-H3ac ChIP experiments were performed and H3ac enrichment at selected region in SNIP1 knockdown cells was compared to scramble control. Enrichment of H3ac was quantified by qPCR. Data are presented as mean values +/- SD (n=3 independent experiments). Statistical significance was estimated using a two-sided student t-test. **i,** Expression levels of DPF3a target genes were analyzed in the primary ccRCC cells after DPF3 knockdown. Data are presented as mean values +/- SD (n=3 independent experiments). Statistical significance was estimated using a two-sided student t-test. **j**, Knockdown of DPF3 decreases local histone acetylation at specific loci in the primary ccRCC cells. Pan-H3ac ChIP experiments were performed and H3ac enrichment at selected region in DPF3 knockdown cells was compared to siNon control. Enrichment of H3ac was quantified by qPCR. Data are presented as mean values +/- SD (n=3 independent experiments). Statistical significance was estimated using a two-sided student t-test. Source data are provided in the Source Data file.

**Supplementary Table 1. SNIP1 immunoprecipitation followed by mass spectrometry analysis performed in 786-O cells.**

| Gene names | Peptides | | Coverage | | Protein names | Comment |
|---|---|---|---|---|---|---|
| | DPF3a- | DPF3a+ | DPF3a- | DPF3a+ | | |
| ACTL6A (BAF53) | 3 | 6 | 10.5 | 22.4 | Actin-like protein 6A | SWI/SNF components identified in IP-MS |
| ARID1A (BAF250A) | 0 | 13 | 0 | 8.6 | AT-rich interactive domain-containing protein 1A | |
| ARID1B (BAF250B) | 0 | 6 | 0 | 5.8 | AT-rich interactive domain-containing protein 1B | |
| DPF3 (BAF45C) | 0 | 4 | 0 | 13.8 | Zinc finger protein DPF3 | |
| SMARCA2 (BRM) | 4 | 8 | 3.1 | 6.9 | SWI/SNF complex subunit SMARCA2 | |
| SMARCA4 (BRG1) | 5 | 11 | 3.3 | 8.5 | SWI/SNF complex subunit SMARCA4 | |
| SMARCB1 (BAF47) | 0 | 4 | 0 | 14.7 | SWI/SNF complex subunit SMARCB1 | |
| SMARCC2 (BAF170) | 3 | 13 | 2.6 | 16.4 | SWI/SNF complex subunit SMARCC2 | |
| SMARCD1 (BAF60A) | 2 | 4 | 4.9 | 8.3 | SWI/SNF complex subunit SMARCD1 | |
| SMARCD2 (BAF60B) | 1 | 4 | 3.3 | 13.6 | SWI/SNF complex subunit SMARCD2 | |
| SMARCE1 (BAF57) | 1 | 7 | 4.7 | 32.5 | SWI/SNF complex subunit SMARCE1 | |
| GSN | 3 | 3 | 5.5 | 5.5 | Gelsolin | SNIP1 interactors independent of DPF3a |
| CCM2 | 7 | 8 | 23.3 | 26.3 | Cerebral cavernous malformations 2 protein | |
| ALB | 5 | 8 | 6.8 | 9.1 | Serum albumin | |
| RDX | 8 | 9 | 13.9 | 15.3 | Radixin | |
| KRIT1 | 4 | 3 | 7.5 | 6.1 | Krev interaction trapped protein 1 | |
| DRG2 | 6 | 6 | 17.8 | 17.8 | Developmentally-regulated GTP-binding protein 2 | |
| CSDE1 | 5 | 6 | 8.5 | 9.9 | Cold shock domain-containing protein E1 | |
| CSNK1D;CSNK1E | 2 | 2 | 5.9 | 5.9 | Casein kinase I isoform delta | |
| OLA1 | 2 | 2 | 5.8 | 5.8 | Obg-like ATPase 1 | |
| RBM42 | 2 | 2 | 10.8 | 10.8 | RNA-binding protein 42 | |
| SDCBP | 2 | 2 | 12.4 | 12.4 | Syntenin-1 | |
| ITGB1BP1 | 2 | 2 | 13.5 | 13.5 | Integrin beta-1-binding protein 1 | |
| NDUFB4 | 2 | 2 | 21.7 | 21.7 | NADH dehydrogenase [ubiquinone] 1 beta subcomplex subunit 4 | |
| PON1 | 2 | 2 | 8.5 | 8.5 | Serum paraoxonase/arylesterase 1 | |
| TRIP10 | 4 | 6 | 7.8 | 12.1 | Cdc42-interacting protein 4 | |
| TFB1M | 2 | 3 | 6.6 | 10.7 | Dimethyladenosine transferase 1, mitochondrial | |
| FAM136A | 2 | 4 | 10.9 | 23.2 | Protein FAM136A | |
| MED18 | 2 | 2 | 11.5 | 11.5 | Mediator of RNA polymerase II transcription subunit 18 | |
| NMNAT1 | 2 | 2 | 10.4 | 10.4 | Nicotinamide/nicotinic acid mononucleotide adenylyltransferase 1 | |
| RCL1 | 2 | 2 | 6.2 | 6.2 | RNA 3-terminal phosphate cyclase-like protein | |
| MRPL11 | 2 | 2 | 15.1 | 15.1 | 39S ribosomal protein L11, mitochondrial | |

**Supplementary Table 2. siRNA and shRNA sequence used in this study.**

| Name | Target | Species | Company | Accession NO. | Target sequence |
|---|---|---|---|---|---|
| siDPF3 | DPF3 | human | Qiagen | NM_012074 | AAGGGAAATCAAAGAATCGA<br>AAGAGGATATTCCCAAGCGAA<br>AACAGACTCTCTGGGCAATTA<br>AAGAAACGTATCAATACCCAT |
| siNon | Non | human | Qingen | | Not provided |
| shSNIP1 | SNIP1 | human | Synthetic | NM_024700 | GCCCGTTAAAGAGAAACCAAG |
| shSNIP1' | SNIP1 | human | Synthetic | NM_024700 | AATTGATCACCCGTCTTGTTC |
| Scramble | Non | human | Synthetic | | CAACAAGATGAAGAGCACCAA |

**Supplementary Table 3. Antibodies. Antibodies used in ChIP, Immunoprecipitation (IP)， Immunofluorescence (IF) and Immunoblotting (IB) and their respective amount used in the experiments are given.**

| Primary antibodies | Company | Used amount/dilution |
|---|---|---|
| Anti-Flag M2, mouse monoclonal | Sigma (F3165) | IB: 1:1000; ChIP: 2μg |
| Anti-HA, rat monoclonal | Roche (11867423001) | IB: 1:2000; IF: 1:500 |
| Anti-HA, rabbit polyclonal | Sigma (H6908) | IP: 2μg; ChIP: 5μg |
| Anti-SNIP1, rabbit polyclonal | Abclonal (A16747) | IF: 1:100; IB: 1:1000; IP: 2μg; ChIP: 5μg |
| Anti-p300, mouse monoclonal | Abcam (ab14984) | IB: 1:1000; ChIP: 5μg |
| Anti-SMAD4, mouse monoclonal | Santa Cruz (sc-7966) | IB: 1:500; ChIP: 5μg |
| Anti-SNAI1, rabbit polyclonal | Abclonal (A5243) | IB: 1:500 |
| Anti-MMP2, rabbit polyclonal | Proteintech (110373-2-AP) | IB: 1:500 |
| Anti-ZEB1, mouse monoclonal | Proteintech (66279-1-Ig) | IB: 1:1000 |
| Anti-ACTB, mouse monoclonal | Proteintech (66009-1-Ig) | IB: 1:10000 |
| Anti-GAPDH, mouse monoclonal | Proteintech (60004-1-Ig) | IB: 1:5000 |
| Anti-H3ac, rabbit polyclonal | Abcam (ab47915) | ChIP: 2μg |
| Normal Mouse IgG | Cell Signaling Technology (5415) | IP: 2μg; ChIP: 5μg |
| Normal Rabbit IgG | Cell Signaling Technology (2729) | IP: 2μg; ChIP: 5μg |
| **Secondary antibodies** | | |
| Rhodamine (TRITC)-conjugated Goat Anti-Rat IgG(H+L) | Proteintech (SA00007-7) | IF: 1:1000 |
| CoraLite488-conjugated Affinipure Goat Anti-Rabbit IgG(H+L) | Proteintech (SA00013-2) | IF: 1: 2000 |
| Peroxidase-conjugated Affinipure Goat Anti-Mouse IgG(H+L) | Proteintech (SA00001-1) | IB: 1:10000 |
| Peroxidase-conjugated Affinipure Goat Anti-Rabbit IgG(H+L) | Proteintech ( SA00001-2) | IB: 1:10000 |
| Anti-Rat IgG-Peroxidase antibody produced in rabbit | Sigma (A9542) | IB: 1:20000 |

**Supplementary Table 4. Sequences of qPCR expression primers and ChIP-qPCR primers.**

| Gene | Forward sequence (5' - 3') | Reverse sequence (5' - 3') | Note |
|---|---|---|---|
| DPF3 | GGCTGCTGGAGATAAAACCTGA | TTCCTGGATGCTTTCCTCCTC | expression |
| DPF3a | GACGATTTGGAAGAGCCTCG | GAGTCTGTTCCGTGGGTTTAGC | expression |
| DPF3b | CGAGGCTGTCAAGACCTACAAG | CGCAGAAGAGTAGCTGGTCATC | expression |
| SNIP1 | CCTCACCACTCAACAGTCAAA | CTCCTGTGTTCCTGTTCTGATG | expression |
| SNAI1 | TCGGAAGCCTAACTACAGCGA | AGATGAGCATTGGCAGCGAG | expression |
| MMP1 | CTCTGGAGTAATGTCACACCTCT | TGTTGGTCCACCTTTCATCTTC | expression |
| MMP2 | CCCACTGCGGTTTTCTCGAAT | CAAAGGGGTATCCATCGCCAT | expression |
| MMP16 | ATGCAGCAGTTCTATGGCATT | CTGGTCAGGTACACCGCATC | expression |
| ZEB1 | TTACACCTTTGCATACAGAACCC | TTTACGATTACACCCAGACTGC | expression |
| WNT7B | CACAGAAACTTTCGCAAGTGG | GTACTGGCACTCGTTGATGC | expression |
| COL1A1 | GAGGGCCAAGACGAAGACATC | CAGATCACGTCATCGCACAAC | expression |
| COL6A3 | TGGGCATTGGCAGGAAGGTG | TCCTCGTTGAGCTCGGTGGA | expression |
| GAPDH | ACCCACTCCTCCACCTTTGA | TTGCTGTAGCCAAATTCGTTG | expression |
| SNAI1 | CGAGTGGTTCTTCTGCGCTA | CCTCCAACGCACCTGGATTA | ChIP-qPCR |
| MMP2 | TATCCCAGGCCTGCCCATGT | GAGCTGGTGGGTGGAAAGCC | ChIP-qPCR |
| COL1A1 | TGGCCAGGGAGGCTGTAACT | GGAAGCCACCGCGGACTAAA | ChIP-qPCR |
| WNT7B | GGCAACCTGAACACGCCTGA | GCCAGCCACAGTCCCAGAAG | ChIP-qPCR |
| COL6A3 | CTCTGTGTCCAGCCCACTGC | CGATGGCATCCCGGACCTG | ChIP-qPCR |
| BAHCC1 | AGCGCCTTGTTTACACTCGGT | CATTCCGGCAGGGACACGAA | ChIP-qPCR |
| HES1 | CCCGGACACACACACACACA | CTGTGCTCGAGCCTGGGAAA | ChIP-qPCR |
| MMP16 | GGCGGTTCCGGCTCAAAGAG | AGGCAGTGCAGGAGGAGGAC | ChIP-qPCR |
| GAPDH | CCGAGCCTCCTTCCTCTCCA | GGCTCCTGGCATCTCTGGGA | ChIP-qPCR |
| IgH | GTCACGGTCAGTGCTGTGCT | CGCCTCCCTATGTGGTGCTG | ChIP-qPCR |
| ACTB | CCTCCGCCCGGTTCAAACA | CTAACTGCGCGTGCGTTCTG | ChIP-qPCR |
