# Supplementary material for: The SWI/SNF chromatin remodeling factor DPF3 regulates metastasis of ccRCC by modulating TGF-β signaling
Source: Nat Commun. 2022 Aug 9;13:4680. doi: 10.1038/s41467-022-32472-0 (PMC9363427; doi:10.1038/s41467-022-32472-0)
Supplement: Supplementary file 3 — Description of Additional Supplementary Files [file 41467_2022_32472_MOESM3_ESM.pdf]

Description of Additional Supplementary Files

File Name: Supplementary Data 1
Description: Differentially expressed genes after DPF3a overexpression in 786-O cells.

File Name: Supplementary Data 2
Description: Differentially expressed genes after SNIP1 knockdown in 786-O cells.

File Name: Supplementary Data 3
Description: Differentially spliced genes upon DPF3a overexpression and SNIP1 knockdown in 786-O cells.
