# Supplementary material for: The SWI/SNF chromatin remodeling factor DPF3 regulates metastasis of ccRCC by modulating TGF-β signaling
Source: Nat Commun. 2022 Aug 9;13:4680. doi: 10.1038/s41467-022-32472-0 (PMC9363427; doi:10.1038/s41467-022-32472-0)
Supplement: Supplementary file 7 — Reporting Summary [file 41467_2022_32472_MOESM7_ESM.pdf]

nature portfolio

Corresponding author(s): Wei Chen

Last updated by author(s): Jul 8, 2022

Reporting Summary

Nature Portfolio wishes to improve the reproducibility of the work that we publish. This form provides structure for consistency and transparency in reporting. For further information on Nature Portfolio policies, see our Editorial Policies and the Editorial Policy Checklist.

Statistics

For all statistical analyses, confirm that the following items are present in the figure legend, table legend, main text, or Methods section.

n/a	Confirmed	
☐	☒	The exact sample size (*n*) for each experimental group/condition, given as a discrete number and unit of measurement
☐	☒	A statement on whether measurements were taken from distinct samples or whether the same sample was measured repeatedly
☐	☒	The statistical test(s) used AND whether they are one- or two-sided<br>*Only common tests should be described solely by name; describe more complex techniques in the Methods section.*
☒	☐	A description of all covariates tested
☐	☒	A description of any assumptions or corrections, such as tests of normality and adjustment for multiple comparisons
☐	☒	A full description of the statistical parameters including central tendency (e.g. means) or other basic estimates (e.g. regression coefficient) AND variation (e.g. standard deviation) or associated estimates of uncertainty (e.g. confidence intervals)
☐	☒	For null hypothesis testing, the test statistic (e.g. *F*, *t*, *r*) with confidence intervals, effect sizes, degrees of freedom and *P* value noted<br>*Give P values as exact values whenever suitable.*
☒	☐	For Bayesian analysis, information on the choice of priors and Markov chain Monte Carlo settings
☒	☐	For hierarchical and complex designs, identification of the appropriate level for tests and full reporting of outcomes
☐	☒	Estimates of effect sizes (e.g. Cohen's *d*, Pearson's *r*), indicating how they were calculated

Our web collection on statistics for biologists contains articles on many of the points above.

Software and code

Policy information about availability of computer code

Data collection	Sequencing data collection was performed on illumina NovaSeq; Proteomics data collection was perform on an LTQ-Orbitrap Elite mass spectrometer system (Thermo).
Data analysis	IGV (v2.5.3); fastQC(v0.11.2); cutadapt (v1.14); HISAT2 (v2.1.0); featureCounts (v2.0.1); DESeq2 (1.28.1); deepTools (v3.5.1); Bowtie2 (v2.3.5.1); MACS2 (v2.2.5); BEDTools (v2.30.0); HOMER (v4.11); fastp(v0.20.1); STAR (version 2.7.8a); rmats (version 4.1.1); MaxQuant (version 1.6.10.0); ImageJ(version 1.8.0); IPA(Qiagen)

For manuscripts utilizing custom algorithms or software that are central to the research but not yet described in published literature, software must be made available to editors and reviewers. We strongly encourage code deposition in a community repository (e.g. GitHub). See the Nature Portfolio guidelines for submitting code & software for further information.

Data

Policy information about availability of data

All manuscripts must include a data availability statement. This statement should provide the following information, where applicable:

- Accession codes, unique identifiers, or web links for publicly available datasets
- A description of any restrictions on data availability
- For clinical datasets or third party data, please ensure that the statement adheres to our policy

All the sequencing data generated from this study have been submitted to the NCBI under the accession number GSE175848 (https://www.ncbi.nlm.nih.gov/geo/query/acc.cgi?acc=GSE175848). The proteomics data have been deposited to the ProteomeXchange Consortium via the PRIDE partner repository with the dataset identifier PXD033421 (http://proteomecentral.proteomexchange.org/cgi/GetDataset?ID=PXD033421). Source data are provided with this paper. The remaining data are available within the Article, Supplementary Information or Source Data file.

Field-specific reporting

Please select the one below that is the best fit for your research. If you are not sure, read the appropriate sections before making your selection.

- [x] Life sciences
- [ ] Behavioural & social sciences
- [ ] Ecological, evolutionary & environmental sciences

For a reference copy of the document with all sections, see nature.com/documents/nr-reporting-summary-flat.pdf

Life sciences study design

All studies must disclose on these points even when the disclosure is negative.

Sample size	No statistical methods were used to predetermine sample size
Data exclusions	No data was excluded from this study.
Replication	Experiments were repeated and our data are based on at least two to three independent experiments with similar results. The precise number of repeats are given in the figure legend.
Randomization	For animal study, 8 weeks old B-NDG female mice were randomized into groups, with 4 mice in each group. For western blot, qPCR, CoIP, GST pulldown, IP-MS, RNA-seq, ChIP-seq, ATAC-seq, MPP2 assay and HAT assay, randomization was not relevant for controlled samples.
Blinding	Blinding was not used in this study, as it was not possible to blind investigator for experiments with controlled samples, including western blot, qPCR, CoIP, GST pulldown, IP-MS, RNA-seq, ChIP-seq, ATAC-seq, MPP2 assay, HAT assay as well as animal study.

Reporting for specific materials, systems and methods

We require information from authors about some types of materials, experimental systems and methods used in many studies. Here, indicate whether each material, system or method listed is relevant to your study. If you are not sure if a list item applies to your research, read the appropriate section before selecting a response.

Materials & experimental systems

n/a	Involved in the study	
☐	☒	Antibodies
☐	☒	Eukaryotic cell lines
☒	☐	Palaeontology and archaeology
☐	☒	Animals and other organisms
☒	☐	Human research participants
☒	☐	Clinical data
☒	☐	Dual use research of concern

Methods

n/a	Involved in the study	
☐	☒	ChIP-seq
☒	☐	Flow cytometry
☒	☐	MRI-based neuroimaging

Antibodies

Antibodies used

Anti-Flag M2, mouse monoclonal, Sigma (F3165), WB: 1:1000, ChIP: 2µg
Anti-HA, rat monoclonal, Roche (11867423001), WB: 1:2000, IF: 1:500
Anti-HA, rabbit polyclonal, Sigma (H6908), IP: 2µg; ChIP: 5µg
Anti-SNIP1, rabbit polyclonal, Abclonal (A16747), IF: 1:100; WB: 1:1000; IP: 2µg; ChIP: 5µg
Anti-p300, mouse monoclonal, Abcam (ab14984), WB: 1:1000; ChIP: 5µg
Anti-SNAI1, rabbit polyclonal, Abclonal (A5243) WB: 1:500
Anti-MMP2, rabbit polyclonal, Proteintech (110373-2-AP), WB: 1:500
Anti-ZEB1, mouse monoclonal, Proteintech (66279-1-Ig), WB: 1:1000
Anti-ACTB, mouse monoclonal, Proteintech (66009-1-Ig), WB: 1:10000
Anti-SMAD4, mouse monoclonal, Santa Cruz (sc-7966), WB: 1:500; ChIP: 5µg
Anti-GAPDH, mouse monoclonal, Proteintech (60004-1-Ig), WB: 1:5000
Anti-H3ac, rabbit polyclonal, Abcam (ab47915), ChIP: 2µg
Normal Mouse IgG, Cell Signaling Technology (5415), IP: 2µg, ChIP: 5µg
Normal Rabbit IgG, Cell Signaling Technology (2729), IP: 2µg, ChIP: 5µg
Rhodamine (TRITC)-conjugated Goat Anti-Rat IgG(H+L); Proteintech (SA00007-7); IF: 1:1000
CoraLite488-conjugated Affinipure Goat Anti-Rabbit IgG(H+L); Proteintech (SA00013-2); IF: 1: 2000
Peroxidase-conjugated Affinipure Goat Anti-Mouse IgG(H+L); Proteintech (SA00001-1); IB: 1:10000
Peroxidase-conjugated Affinipure Goat Anti-Rabbit IgG(H+L); Proteintech (SA00001-2); IB: 1:10000
Anti-Rat IgG-Peroxidase antibody produced in rabbit; Sigma (A9542); IB: 1:20000
Biotinylated anti-TGFB1 antibody (provided in the ELISA kit), Abcam (ab100647); ELISA: 1:80

Validation	Flag M2 (Sigma, F3165). Tested applications: WB, IP, IHC, IF, ELISA, EIA, ChIP, EM, Flow cytometry. 6773 references on the manufacturer's website.<br>HA (Roche, 11867423001). Tested applications: Dot blots, ELISA, IHC, IP, WB. 24 references on the manufacturer's website.<br>HA (Sigma, H6908). Tested applications: IP, WB, IHC. 904 references on the manufacturer's website.<br>SNIP1 (Abclonal, A16747). Tested reactivity: Human, Rat. Tested applications: WB, IHC, IF. We have validated this antibody in knockdown and overexpression experiments.<br>p300 (Abcam, ab14984). Tested reactivity: Human. Tested applications: WB, ChIP. 62 references on the manufacturer's website.<br>SNAI1 (Abclonal, A5243). Tested reactivity: Human, Mouse, Rat. Tested applications: WB, IHC, IF. 26 references on the manufacturer's website.<br>MMP2 (Proteintech, 110373-2-AP). Tested reactivity: Human. Tested applications: WB, IP, IHC, IF, ELISA. 585 references on the manufacturer's website.<br>ZEB1 (Proteintech, 66279-1-Ig). Tested reactivity: Human. Tested applications: WB, IHC. 3 references on the manufacturer's website.<br>ACTB (Proteintech, 66009-1-Ig). Tested reactivity: Human, Mouse, Rat, Hamster, Zebrafish, Monkey, Dog. Tested applications: WB, IP, IHC, IF, FC, CoIP, ChIP, ELISA. 3164 references on the manufacturer's website.<br>SMAD4 (Santa Cruz, sc-7966). Tested reactivity: Human, Mouse, Rat. Tested applications: WB, IP, IF, IHC, IP, FCM, ELISA. 762 references on the manufacturer's website.<br>GAPDH (Proteintech, 60004-1-Ig). Tested reactivity: Human, Mouse, Rat, Yeast, Plant, Zebrafish. Tested applications: WB, IP, IHC, IF, FC, CoIP, ChIP, ELISA. 6073 references on the manufacturer's website.<br>H3ac (Abcam, ab47915). Tested reactivity: Human. Tested applications: ChIP, Dot blot, WB. 96 references on the manufacturer's website.

Eukaryotic cell lines

Policy information about cell lines

Cell line source(s)	The 786-O (CRL-1932) and HEK293T (CRL-3216) cells were obtained from the ATCC. The primary cells isolated from primary tumors of ccRCC patients were obtained from Dr. Walter Birchmeier' Lab at Max-Delbrueck-Center for Molecular Medicine, Berlin.
Authentication	None of the cells were authenticated.
Mycoplasma contamination	Cell lines tested negative for mycoplasma.
Commonly misidentified lines (See ICLAC register)	No commonly misidentified cells lines were used in this study.

Animals and other organisms

Policy information about studies involving animals; ARRIVE guidelines recommended for reporting animal research

Laboratory animals	B-BNG mice (stain name: NOD.CB17-PrkdcscidIl2rgtm1/Bcgen), 8 weeks old female.
Wild animals	This study did not involve wild animals.
Field-collected samples	This study did not involve samples collected from the field.
Ethics oversight	All animal experiments were carried out following animal protocols approved by the Laboratory Animal Welfare and Ethics Committee of Southern University of Science and Technology.

Note that full information on the approval of the study protocol must also be provided in the manuscript.

ChIP-seq

Data deposition

- [x] Confirm that both raw and final processed data have been deposited in a public database such as GEO.
- [x] Confirm that you have deposited or provided access to graph files (e.g. BED files) for the called peaks.

Data access links<br>*May remain private before publication.*	https://www.ncbi.nlm.nih.gov/geo/query/acc.cgi?acc=GSE175848<br>token wjqfwusyftenvmr
Files in database submission	HA-DPF3a_ChIP-seq_rep1_1.fq.gz<br>HA-DPF3a_ChIP-seq_rep1_2.fq.gz<br>HA-DPF3a_ChIP-seq_rep2_1.fq.gz<br>HA-DPF3a_ChIP-seq_rep2_2.fq.gz<br>SMAD4_ChIP-seq_rep1_1.fq.gz<br>SMAD4_ChIP-seq_rep1_2.fq.gz<br>SMAD4_ChIP-seq_rep2_1.fq.gz<br>SMAD4_ChIP-seq_rep2_2.fq.gz<br>SNIP1_ChIP-seq_rep1_1.fq.gz

SNIP1_ChIP-seq_rep1_2.fq.gz<br>SNIP1_ChIP-seq_rep2_1.fq.gz<br>SNIP1_ChIP-seq_rep2_2.fq.gz<br>p300_ChIP-seq_rep1_1.gz<br>p300_ChIP-seq_rep1_2.gz<br>p300_ChIP-seq_rep2_1.gz<br>p300_ChIP-seq_rep2_2.gz<br>HA-DPF3a_ChIP-seq_rep1.bw<br>HA-DPF3a_ChIP-seq_rep2.bw<br>SMAD4_ChIP-seq_rep1.bw<br>SMAD4_ChIP-seq_rep2.bw<br>SNIP1_ChIP-seq_rep1.bw<br>SNIP1_ChIP-seq_rep2.bw<br>p300_ChIP-seq_rep1.bw<br>p300_ChIP-seq_rep2.bw<br>DPF3a_peaks_50.narrowPeak<br>P300_peaks_50.narrowPeak<br>SMAD4_peaks_50.narrowPeak<br>SNIP1_peaks_50.narrowPeak

Genome browser session<br>(e.g. UCSC)	All individual replicate and bw files have been uploaded to GEO:<br>https://www.ncbi.nlm.nih.gov/geo/query/acc.cgi?acc=GSE175848<br>token wjqfwusyftenvmr<br>Files can be downloaded and viewed with IGV.

Methodology

Replicates	All ChIP-seq were performed in two biological replicates.
Sequencing depth	ChIP-seq<br>Sample #reads, deduplicated, MAPQ > 30, excluding mapped to mitochondrial genome<br>DPF3a_rep1.dup.sort.bam 11325118<br>DPF3a_rep2.dup.sort.bam 20743282<br>SNIP1_rep1.dup.sort.bam 31133972<br>SNIP1_rep2.dup.sort.bam 21806122<br>SMAD4_rep1.dup.sort.bam 40919672<br>SMAD4_rep2.dup.sort.bam 21132788<br>P300_rep1.dup.sort.bam 27906176<br>P300_rep2.dup.sort.bam 27794770
Antibodies	Listed above.
Peak calling parameters	macs2 callpeak --nomodel -g hs -f BAM
Data quality	Sample #peaks with FDR < 1e-5<br>DPF3a_peaks_50.narrowPeak 51530<br>SNIP1_peaks_50.narrowPeak 22791<br>SMAD4_peaks_50.narrowPeak 46367<br>P300_peaks_50.narrowPeak 20380
Software	To analyze ChIP-seq data, the sequencing reads were mapped to hg38 reference genome by Bowtie2 (version 2.3.5.1) after quality control and adapter trimming (same as above). The reads with MAPQ lower than 30 or mapped to mitochondria were removed for further analysis. After duplicate removal by Picard tools (http://broadinstitute.github.io/picard), peaks were called using MACS2 (version 2.2.5) with the parameters -g hs. Peaks with strong signals (peak integer score > 50, i.e. int(-10*log10qvalue) > 50) were retained for further analysis.
